# Evaluation of the use of dienogest in women with deep endometriosis and ovarian endometrioma: a retrospective cohort study

**DOI:** 10.1590/1806-9282.20260113

**Published:** 2026-06-22

**Authors:** Arissa Onishi, Joao Paulo Leonardo-Pinto, Cristina Laguna Benetti-Pinto, Daniela Angerame Yela

**Affiliations:** 1 Universidade Estadual de Campinas, School of Medical Sciences, Department of Gynecology and Obstetrics – Campinas (SP), Brazil.

**Keywords:** Endometriosis, Endometrioma, Pelvic pain, Dysmenorrhea

## Abstract

**OBJECTIVE:**

The aim of this study was to evaluate the use of dienogest in the treatment of deep endometriosis and ovarian endometrioma.

**METHODS:**

This retrospective cohort study included 59 women diagnosed with ovarian endometrioma at a tertiary hospital between 2013 and 2018. Pain scores and endometrioma size were evaluated after 12 months of dienogest use, along with the women's sociodemographic characteristics.

**RESULTS:**

The mean age of the participants was 35.7±6.9 years. Unilateral endometrioma was observed in 38.9% of cases. There was a significant reduction in dysmenorrhea (p=0.011) with dienogest use, but no reduction in other pain symptoms. A reduction in left ovarian volume (p=0.009), mean left endometrioma size (p=0.01), and lesion size in the anterior cul-de-sac (p=0.047) was observed after dienogest treatment. A positive correlation was found between dyschezia and lesions in the posterior cul-de-sac before initiation of dienogest treatment.

**CONCLUSION:**

Dienogest appears to reduce pain in women with deep endometriosis. Our findings support dienogest as an effective therapeutic option.

## INTRODUCTION

Endometriosis is a disease characterized by the presence of endometrial tissue, glands, and/or stroma outside the uterus. It commonly presents with dysmenorrhea, dyspareunia, chronic pelvic pain, and infertility, making it a debilitating condition that can significantly impair the quality of life of affected women^1^. Up to 67% of women with endometriosis may develop endometriomas^2,3^.

Treatment for endometriosis may be either medical or surgical. The choice of management should be individualized according to each woman's specific characteristics^4^.

The primary goal of surgery for endometriosis is to remove lesions and relieve pain. Surgical techniques for ovarian endometriosis typically include cystectomy, cyst puncture or fenestration, and cauterization of the cyst wall^5^. The impact on oocyte quality has been little studied; however, the literature suggests that ovarian endometrioma negatively affects both the quality and quantity of oocytes^6^. On the other hand, surgery is associated with decreased ovarian reserve, a considerable risk of recurrence, and possible tubal obstruction, which may cause irreversible damage. Moreover, surgical complications may delay subsequent fertility treatment^7^.

Although cystectomy has been considered the first-line surgical treatment for ovarian endometriomas, it may have a detrimental effect on ovarian reserve. Therefore, alternative techniques, such as CO_2_ laser vaporization, should be considered^8^.

Given that endometriosis is a chronic disease that can cause persistent symptoms and carries a risk of recurrence, particularly in women of reproductive age, medical management has gained increasing prominence in pain control. Among medical treatments, progestogens are considered first-line therapy. A systematic review did not demonstrate superiority in pain control among different progestogen options or combined oral contraceptives^9^. Dienogest, a fourth-generation progestogen, acts by inducing endometrial decidualization through relative inhibition of gonadotropin secretion and consequent reduction in endogenous estradiol production. It also reduces the responsiveness of ectopic endometrial tissue to endogenous estradiol by modulating the expression of metalloproteinases and aromatase^10^.

Dienogest has been shown to be effective in reducing pain symptoms associated with endometriosis and in improving quality-of-life indices reported by patients^11^. Furthermore, some studies have demonstrated that dienogest is associated with a reduction in endometrioma volume^12–16^. The exact mechanism underlying this effect has not yet been fully elucidated; however, it is likely related to alterations in gene expression, reduction of the local inflammatory response, and inhibition of angiogenesis^3,16,17^.

Therefore, the present study aims to evaluate the treatment of deep endometriosis and ovarian endometrioma with dienogest.

## METHODS

This retrospective cohort study included 59 women with ovarian endometrioma and deep endometriosis who were followed at the endometriosis clinic of a tertiary hospital between 2013 and 2018. Women of reproductive age with an ultrasound diagnosis of ovarian endometrioma who had been using 2 mg of dienogest daily for at least 12 months were eligible for inclusion. Women were excluded if they had incomplete medical records, discontinued or changed the medication before completing the proposed treatment period, or underwent surgery before completing 12 months of clinical treatment.

The variables analyzed included age, race (white or non-white), marital status (with or without a partner), parity, body mass index (BMI), pain symptoms (dysmenorrhea, dyspareunia, chronic pelvic pain, dyschezia, and dysuria), previous surgeries, comorbidities, and ultrasound findings (uterine volume and ovarian volume). The presence of endometriotic lesions (uterovesical region, rectovaginal septum, bowel, bladder, ovarian endometrioma, and adenomyosis), the size of the ovarian endometrioma (in millimeters), and the number of endometriotic lesions were also recorded.

All women developed amenorrhea during treatment; however, some reported persistent spotting.

Pain symptoms were assessed using a 10-point visual analog scale (VAS), ranging from 0 (no pain) to 10 (worst pain imaginable). Pain was classified as mild (scores 1–3), moderate (scores 4–7), or severe (scores >7)^18^.

Ultrasound examinations were performed by a single researcher with more than ten years of experience, using Toshiba X (Aryan, Spain) or Voluson E8 (GE Healthcare, Austria) equipment. All examinations were conducted after bowel preparation. Lesions were described in millimeters, and lesion volume was calculated by multiplying three orthogonal measurements (depth, length, and width) and applying a correction factor of 0.52. An anatomical description of the lesion location was also provided.

Ultrasound examinations were performed systematically, with each endometriotic lesion described and measured according to the consensus statement of the International Deep Endometriosis Analysis group. For ovarian endometriomas, the largest diameter (in millimeters) was used for analysis. The largest diameter was also considered for lesions in the uterovesical region, rectovaginal septum, bowel, and bladder.

This study was approved by the institution's Research Ethics Committee (protocol number: 44360621.8.0000.5404). All items of the STROBE (Strengthening the Reporting of Observational studies in Epidemiology) checklist for cohort studies were followed.

### Statistical analysis

Frequencies, means, and standard deviations were calculated for the study variables. The McNemar test was used to compare paired categorical variables, and the Wilcoxon signed-rank test was used for paired numerical variables. Spearman's correlation coefficient was applied to assess the relationship between pain symptoms and ultrasound findings. A significance level of 5% was adopted for all statistical tests. Statistical analyses were performed using Statistical Analysis System, version 9.4.

The sample size calculation was performed to compare pain scores and endometrioma volume before and after treatment with dienogest, based on estimates from the literature^17^. Considering a significance level of 5% and statistical power of 80%, the minimum required sample size was estimated at 49 women.

## RESULTS

A total of 841 medical records were reviewed. Of these, 782 were excluded due to the absence of documented endometrioma on the initial ultrasound, lack of follow-up imaging after treatment, use of other medications for endometriosis, irregular use of dienogest or use for less than 12 months, or surgery performed before completing 12 months of clinical treatment.

Thus, 59 women aged 19–51 years were included (mean age 35.7±6.9 years), with a mean BMI of 28.1±5.7 kg/m^2^; 79.0% were white. Additionally, 39.5% had undergone previous surgery. Unilateral endometrioma was observed in 38.9% of the participants, with most cases occurring in the right ovary. Lesions in the uterovesical region were present in 30.5% of women, whereas 71.1% had lesions in the rectovaginal septum and 77.9% had bowel involvement (Table 1).

**Table 1:** Clinical and sociodemographic characteristics and ultrasound characteristics of women with ovarian endometrioma (n=59).

Variable	Mean±SD/n (%)
Age (years)	35.7±6.9
Nulliparous	28 (49.1)
Withe	47 (79.6)
With partner	27 (79.4)
BMI (kg/m^2^)	28.1±5.7
Previous surgeries	19 (39.5)
Comorbidities	21 (55.2)
Uterine volume (mm^3^)	103.5±57.4
Right ovarian volume (mm^3^)	31.6±77.6
Left ovarian volume (mm^3^)	27.7±44.3
Unilateral endometrioma	23 (38.9)
Adenomyosis	14 (23.7)
Uterovesical region lesion	18 (30.5)
Rectovaginal septum lesion	42 (71.1)
Bowel lesion	46 (77.9)

SD: standard deviation; BMI: body mass index; mm^3^: cubic millimeters.

A statistically significant reduction in dysmenorrhea was observed after 12 months of treatment (p=0.011); however, no significant improvement was noted in other pain symptoms. There was no difference in the number of endometriomas in either ovary (p=1.00).

Regarding ultrasound findings, mean right ovarian volume increased (p=0.021), whereas mean left ovarian volume decreased (p=0.009). The mean diameter of the right ovarian endometrioma remained unchanged (p=0.635), while a significant reduction was observed in the left ovary (p=0.01). Overall, there was a 7% reduction in left ovarian volume and an 11% reduction in the largest diameter of left ovarian endometriomas. A reduction in lesion size in the uterovesical region was also observed (p=0.047) (Table 2).

**Table 2:** Assessment of pain symptoms ultrasound results of women with endometriosis using dienogest for 12 months (n=59).

	Initial Mean±SD	12 months Mean±SD	p
Dysmenorrhea	3.1±4.0	1.8±3.3	0.011
Chronic pelvic pain	3.2±3.7	2.2±3.2	0.136
Dyspareunia	1.9±3.1	2.2±3.4	0.579
Dyschezia	1.7±2.8	1.5±3.0	0.685
Dysuria	0.6±2.1	0.6±2.1	0.813
Right ovarian volume (mm^3^)	23.9±51.8	42.3±211.7	0.021
Left ovarian volume (mm^3^)	28.4±44.9	26.4±55.8	0.009
Right endometrioma (mm)	26.1±17.7	28.8±33.0	0.635
Left endometrioma (mm)	30.5±18.7	27.2±22.1	0.01
Right endometrioma number	1.2±0.4	1.2±0.4	1.00
Left endometrioma number	1.1±0.3	1.1±0.4	1.00
Uterovesical region lesion (mm)	29.7±7.4	24.7±7.4	0.047
Rectovaginal septum lesion (mm)	35.8±7.7	33.1±9.7	0.287
Bowel lesion (mm)	26.5±12.7	26.8±12.8	0.706

SD: standard deviation; mm: millimeters; Wilcoxon test.

No correlation was found between dysmenorrhea or dyspareunia and lesion size at any anatomical location. In general, no strong correlations were identified between symptoms and lesion size; however, some weak associations were observed. A weak positive correlation was found between chronic pelvic pain and the initial size of the lesion in the uterovesical region (r=0.49; p=0.04), as well as between dyschezia and the initial size of the lesion in the rectovaginal septum (r=0.39; p=0.01). A weak negative correlation was observed between dyschezia and the initial size of the endometrioma (r=-0.31; p=0.03), and between dysuria and the final size of the lesion in the rectovaginal septum (r=-0.41; p=0.01) (Table 3).

**Table 3:** Correlation of pain symptoms with endometriosis lesions before and after treatment with dienogest (n=59).

		Initial—lesions				12 months—lesions			
		Endometrioma	Uterovesical region	Rectovaginal septum	Bowel	Endometrioma	Uterovesical region	Rectovaginal septum	Bowel
Dysmenorrhea									
	r	0.05	0.17	0.26	0.26	0.14	-0.03	0.003	0.03
	p	0.71	0.51	0.11	0.09	0.41	0.87	0.98	0.81
Chronic pelvic pain									
	r	-0.02	0.49	0.10	0.13	0.18	-0.007	0.05	0.13
	p	0.88	0.04	0.51	0.39	0.29	0.97	0.76	0.39
Dyspareunia									
	r	0.02	0.25	0.12	-0.02	-0.23	0.06	0.16	-0.11
	p	0.87	0.36	0.47	0.88	0.22	0.79	0.41	0.50
Dyschezia									
	r	-0.31	0.47	0.39	0.14	-0.05	-0.01	-0.09	0.007
	p	0.03	0.05	0.01	0.35	0.75	0.95	0.60	0.95
Dysuria									
	r	-0.06	-0.09	0.19	0.17	0.01	0.00	-0.41	0.13
	p	0.70	0.73	0.26	0.27	0.94	1.00	0.01	0.39

r: Spearman correlation coefficient.

## DISCUSSION

A significant reduction in dysmenorrhea was observed with the use of dienogest; however, no improvement was noted in other pain symptoms. Additionally, a reduction in left ovarian volume and in the mean size of the left ovarian endometrioma and uterovesical lesions was observed after treatment. A positive correlation between dyschezia and rectovaginal septum lesions was identified before initiation of dienogest therapy.

A statistically significant reduction (p<0.01) in dysmenorrhea intensity was observed, with an approximate 54.4% decrease in the mean VAS score. This finding is consistent with previous studies. Gokmen et al. reported a 37.6% reduction in mean VAS scores^12^. Another retrospective study including 297 women demonstrated that all treatment groups showed a significant reduction in endometrioma size after 12 months, with the group receiving dienogest alone experiencing significant improvement in dysmenorrhea^13^.

In our study, a 3.3 mm (10%) reduction in the mean largest diameter of the endometrioma was observed (p=0.01). Progressive reduction in endometrioma size during dienogest treatment has been described in previous studies, although there is heterogeneity in measurement methods across the literature. Gokmen et al. demonstrated a significant decrease in mean endometrioma size from 44.0±13.0 mm at baseline to 39.5±15.0 mm after 3 months and 34.4±18.0 mm after 6 months^12^.

Similarly, Forno et al. reported a reduction of 2.5 mm after 6 months and 6.5 mm after 12 months in the mean endometrioma diameter^13^. Another study evaluating the largest endometrioma dimension demonstrated a 30% reduction after 3 months of dienogest use^15^. Furthermore, a study reported a decrease in the average diameter of ovarian endometriomas after 12 months of treatment, with a consequent 30% reduction in the need for surgical intervention^19^.

Regarding ultrasound parameters, some studies use the volume of the largest endometrioma as the primary outcome to assess therapeutic efficacy. Several reports have demonstrated reductions in endometrioma volume and, in some cases, complete resolution^16,20–22^. A systematic review including 16 studies and 888 women concluded that hormonal therapy may reduce endometrioma size^23^.

The mechanism underlying these findings may be explained by the action of dienogest in reducing endogenous estradiol production, thereby inducing decidualization and subsequent atrophy of ectopic endometrial tissue. In addition, its immunomodulatory and antiangiogenic effects may inhibit cellular proliferation, contributing to lesion reduction^3,17,24^.

In our study, no strong correlation was observed between pain symptoms and lesion size. Similarly, previous research has not consistently demonstrated an association between lesion size and pain severity^25^. The weak positive correlations between pain and initial lesion size may reflect the inflammatory activity present before treatment initiation. A study evaluating dysmenorrhea in women with ovarian endometrioma suggested that greater fibrosis and inflammatory response are associated with increased pain intensity^26^.

This study has several limitations. First, its retrospective design limits causal inference. Second, the inclusion of women who had undergone previous surgery may introduce selection bias, as surgical cases with poor clinical response during the study period were excluded. Additionally, women without follow-up ultrasound examinations were excluded; it is possible that clinical improvement reduced the indication for repeat imaging, potentially introducing further bias. Due to the retrospective nature of the study, medication adherence could not be objectively assessed.

Given these limitations, we cannot definitively conclude that dienogest leads to clinically meaningful reductions in endometrioma size, as the observed mean reduction of approximately 3 mm may not be clinically significant.

Despite these limitations, our findings add to the growing body of evidence supporting the use of dienogest for pain management in women with ovarian endometriomas and deep endometriosis. Its role appears particularly relevant for young women who wish to preserve fertility and avoid surgical intervention.

## CONCLUSION

Our study supports the use of dienogest in the management of pain symptoms associated with ovarian endometriomas and deep endometriosis, particularly in reducing dysmenorrhea. Prospective studies with larger sample sizes and longer follow-up periods are needed to confirm these findings and further clarify the long-term efficacy and safety profile of dienogest in clinical practice.

## Data Availability

The datasets generated and/or analyzed during the current study are available from the corresponding author upon reasonable request.

## References

[1] Giudice LC, Kao LC (2004). Endometriosis. Lancet.

[2] Forno S, Mabrouk M, Arena A, Mattioli G, Giaquinto I, Paradisi R (2019). Dienogest or norethindrone acetate for the treatment of ovarian endometriomas: can we avoid surgery?. Eur J Obstet Gynecol Reprod Biol.

[3] Eberle A, Nguyen DB, Smith JP, Mansour FW, Krishnamurthy S, Zakhari A (2024). Medical management of ovarian endometriomas: a systematic review and meta-analysis. Obstet Gynecol.

[4] Chandra A, Rho AM, Jeong K, Yu T, Jeon JH, Park SY (2018). Clinical experience of long-term use of dienogest after surgery for ovarian endometrioma. Obstet Gynecol Sci.

[5] Fan Y, Yang Q, Lin Y, Fu X, Shu J (2025). The effect of endometriosis on oocyte quality: mechanisms, diagnosis and treatment. Arch Gynecol Obstet.

[6] Wu Y, Yang R, Lan J, Lin H, Jiao X, Zhang Q (2021). Ovarian endometrioma negatively impacts oocyte quality and quantity but not pregnancy outcomes in women undergoing IVF/ICSI treatment: a retrospective cohort study. Front Endocrinol.

[7] Urman B, Muzii L, Ertas S, Aksakal E, Usta I, Seyhan A (2026). Is surgery for endometriomas ever indicated?. Reprod Biomed Online.

[8] Bafort C, Lie Fong S, Fieuws S, Geysenbergh B, Nisolle M, Squifflet JL (2025). Conservative endometrioma surgery: the combined technique versus CO2-laser vaporization only (BLAST: Belgium LAser STudy): clinical protocol for a multicenter randomized controlled trial. PLoS One.

[9] Souza Gaio G, Kemczenski F, Fernandes Oliveira Amador W, Farias CF, Chagas J (2025). Clinical effectiveness of progestogens compared to combined oral contraceptive pills in the treatment of endometriosis: a systematic review and meta-analysis. Eur J Obstet Gynecol Reprod Biol.

[10] Lee J, Park HJ, Yi KW (2023). Dienogest in endometriosis treatment: a narrative literature review. Clin Exp Reprod Med.

[11] Keukens A, Veth VB, Regis M, Mijatovic V, Bongers MY, Coppus SFPJ (2024). The effect of surgery or medication on pain and quality of life in women with endometrioma. A systematic review and meta-analysis. Eur J Obstet Gynecol Reprod Biol.

[12] Gokmen BS, Selcuki NFT, Aydan A, Bahat PY, Akca A (2023). Effects of dienogest therapy on endometriosis-related dysmenorrhea, dyspareunia, and endometrioma size. Cureus.

[13] Forno S, Orsini B, Verrelli L, Caroli M, Aru AC, Lenzi J (2023). Dienogest alone or dienogest combined with estrogens in the treatment of ovarian endometriomas, that is the question. A retrospective cohort study. Arch Gynecol Obstet.

[14] Momoeda M, Harada T, Terakawa N, Aso T, Fukunaga M, Hagino H (2009). Long-term use of dienogest for the treatment of endometriosis. J Obstet Gynaecol Res.

[15] Sugimoto K, Nagata C, Hayashi H, Yanagida S, Okamoto A (2015). Use of dienogest over 53 weeks for the treatment of endometriosis. J Obstet Gynaecol Res.

[16] Angioni S, Pontis A, Malune ME, Cela V, Luisi S, Litta P (2020). Is dienogest the best medical treatment for ovarian endometriomas? Results of a multicentric case control study. Gynecol Endocrinol.

[17] Mueck AO (2011). Dienogest: an oral progestogen for the treatment of endometriosis. Exp Rev Obstet Gynecol.

[18] Jensen MP, Chen C, Brugger AM (2003). Interpretation of visual analog scale ratings and chance scores: a reanalysis of two clinical trials of postoperative pain. J Pain.

[19] Ferrari F, Epis M, Casarin J, Bordi G, Gisone EB, Cattelan C (2024). Long-term therapy with dienogest or other oral cyclic estrogen-progestogen can reduce the need for ovarian endometrioma surgery. Womens Health (Lond).

[20] Xholli A, Fillip G, Previtera F, Cagnacci A (2020). Modification of endometrioma size during hormone therapy containing dienogest. Gynecol Endocrinol.

[21] Uludag SZ, Demirtas E, Sahin Y, Aygen EM (2021). Dienogest reduces endometrioma volume and endometriosis related pain symptoms. J Obstet Gynaecol.

[22] Maiorana A, Maranto M, Restivo V, Gerfo D, Minneci G, Mercurio A (2024). Evaluation of long-term efficacy and safety of dienogest in patients with chronic cyclic pelvic pain associated with endometriosis. Arch Gynecol Obstet.

[23] Thiel PS, Donders F, Kobylianskii A, Maheux-Lacroix S, Matelski J, Walsh C (2024). The effect of hormonal treatment on ovarian endometriomas: a systematic review and meta-analysis. J Minim Invasive Gynecol.

[24] Choi JY, Jo M, Lee E, Lee DY (2020). Dienogest regulates apoptosis, proliferation, and invasiveness of endometriotic cyst stromal cells via endoplasmic reticulum stress induction. Mol Hum Reprod.

[25] Pashkunova D, Darici E, Senft B, Bokor A, Hudelist T, Tammaa A (2024). Lesion size and location in deep infiltrating bowel endometriosis: correlation with gastrointestinal dysfunction and pain. Acta Obstet Gynecol Scand.

[26] Nie J, Zhao C, Laganà AS, Liu X, Guo SW (2022). Identification of lesional attributes of dysmenorrhea severity and the serum antimüllerian hormone levels in women with ovarian endometriomas. Fertil Steril.

